# Infected left atrial myxoma with *Streptococcus gordonii*: case report and literature review

**DOI:** 10.3389/fonc.2025.1635642

**Published:** 2025-10-15

**Authors:** Liqin Ruan, Shixiong Chen, Jing Zhang, Guiping Peng

**Affiliations:** ^1^ Department of Hepatobiliary Surgery, Jiujiang City Key Laboratory of Cell Therapy, Jiujiang NO.1 People’s Hospital, Jiujiang, Jiangxi, China; ^2^ Laboratory of Cardiothoracic Surgery Department, Jiujiang City Key Laboratory of Cell Therapy, Jiujiang NO.1 People’s Hospital, Jiujiang, Jiangxi, China; ^3^ Laboratory of Pathology, Jiujiang City Key Laboratory of Cell Therapy, Jiujiang NO.1 People’s Hospital, Jiujiang, Jiangxi, China; ^4^ Department of Ultrasound, Jiujiang City Key Laboratory of Cell Therapy, Jiujiang NO.1 People’s Hospital, Jiujiang, Jiangxi, China

**Keywords:** infected cardiac myxoma, *Streptococcus gordonii*, transesophageal echocardiography, metagenomic next-generation sequencing, blood cultures

## Abstract

Cardiac myxoma is a relatively common type of benign heart tumor, but infectious myxoma is rare. The symptoms of non-infected cardiac myxoma and infected cardiac myxoma are similar and mostly nonspecific, which can easily lead to delayed diagnosis, missed diagnosis, and delayed treatment. A 57-year-old male patient presented with nonspecific systemic symptoms such as anorexia, fever, and cough, and was initially considered to have gastrointestinal disease or pulmonary infection. Preoperative bacterial culture was negative, and imaging features were consistent with cardiac myxoma. A small amount of vegetation was found attached to the surface of the tumor. Postoperative blood culture, surgical specimen culture, and postoperative blood metagenomic next-generation sequencing (mNGS) examination all showed positive results for *Streptococcus gordonii*, confirming the diagnosis of infectious left atrial myxoma. For patients with febrile cardiac myxoma, it is crucial to be vigilant against concurrent infections. Blood cultures should be performed before administering antibiotics. In cases where blood cultures are negative, a combination of mNGS, PCR, and transesophageal echocardiography (TEE) should be utilized for differential diagnosis, with particular attention paid to the characteristics of vegetations on the tumor surface.

## Background

Cardiac myxomas are uncommon neoplasms. The classic clinical presentation of cardiac myxomas often encompasses a triad of symptoms: systemic, obstructive, and embolic manifestations (1, 2). Infected cardiac myxomas represent an exceedingly rare clinical scenario, with only isolated cases documented in the medical literature (3, 4).

## Case report

A 57-year-old male patient presented to the hospital emergency department with a one-month history of intermittent fever, fatigue of unknown origin, anorexia, occasional cough, expectoration of white sputum, and frequent urination. Initially admitted to the gastroenterology department of our hospital, the patient was suspected of having a digestive tract disease, lung infection, or urinary tract infection. The patient has a history of newly diagnosed type 2 diabetes mellitus and denies any history of dental procedures. On physical examination, his blood pressure was 118/73 mmHg, heart rate was 110 beats per minute, body temperature was 38.2°C, and respiratory rate was 18 breaths per minute. Notably, the first heart sound was intensified, and an occasional soft early diastolic murmur was auscultated. Lung examination revealed coarse breath sounds bilaterally. The abdominal examination was unremarkable, but mild bilateral edema was observed in the extremities. The remaining physical examination findings were normal.

**Figure 1:**
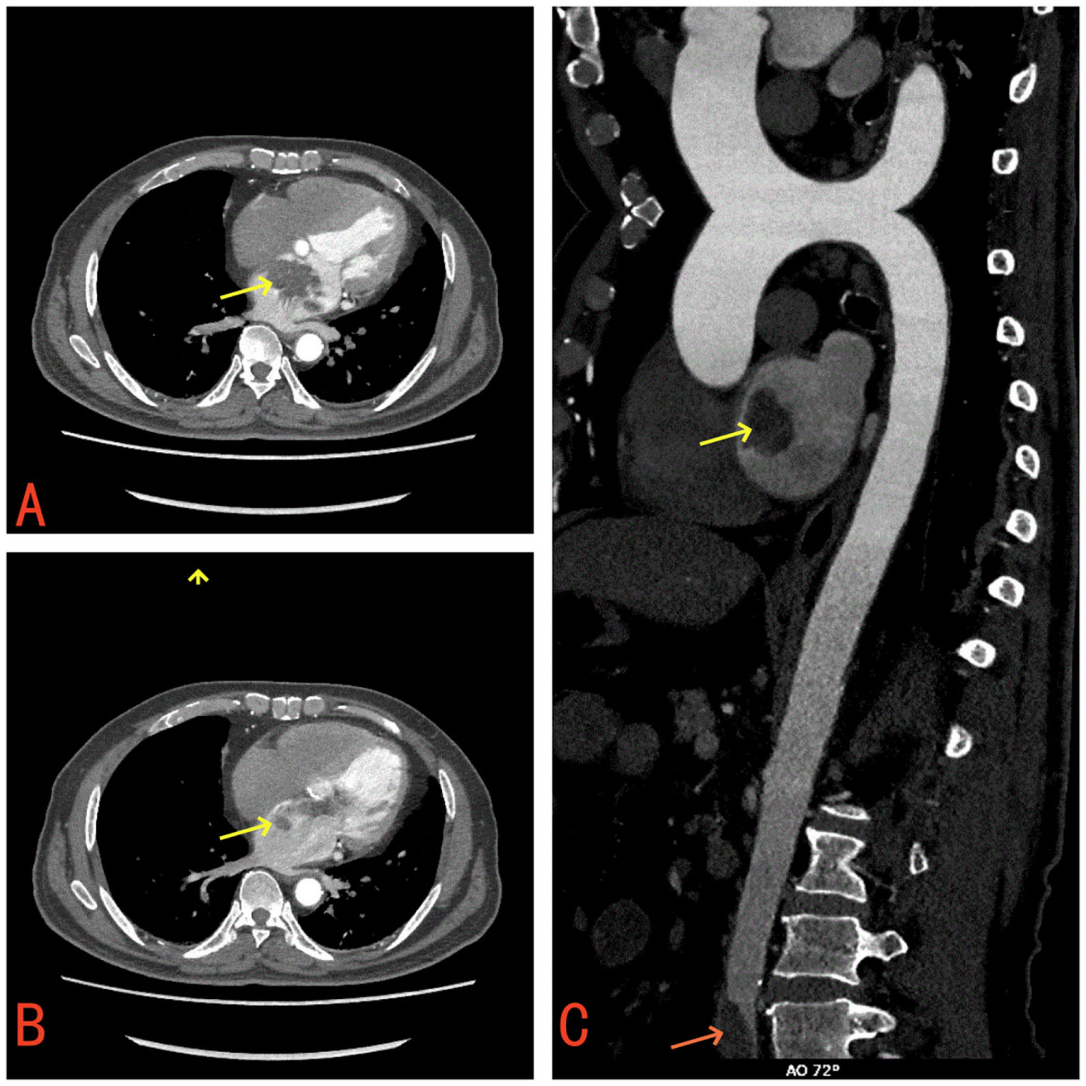
**(A)** Contrast-enhanced CT demonstrates a mass in the left atrium (yellow arrow). **(B)** The left atrial mass prolapses through the mitral valve into the left ventricle (yellow arrow). **(C)** A filling defect suggestive of thrombus observed at the bifurcation of the abdominal aorta and bilateral common iliac arteries (red arrow).

**Figure 2:**
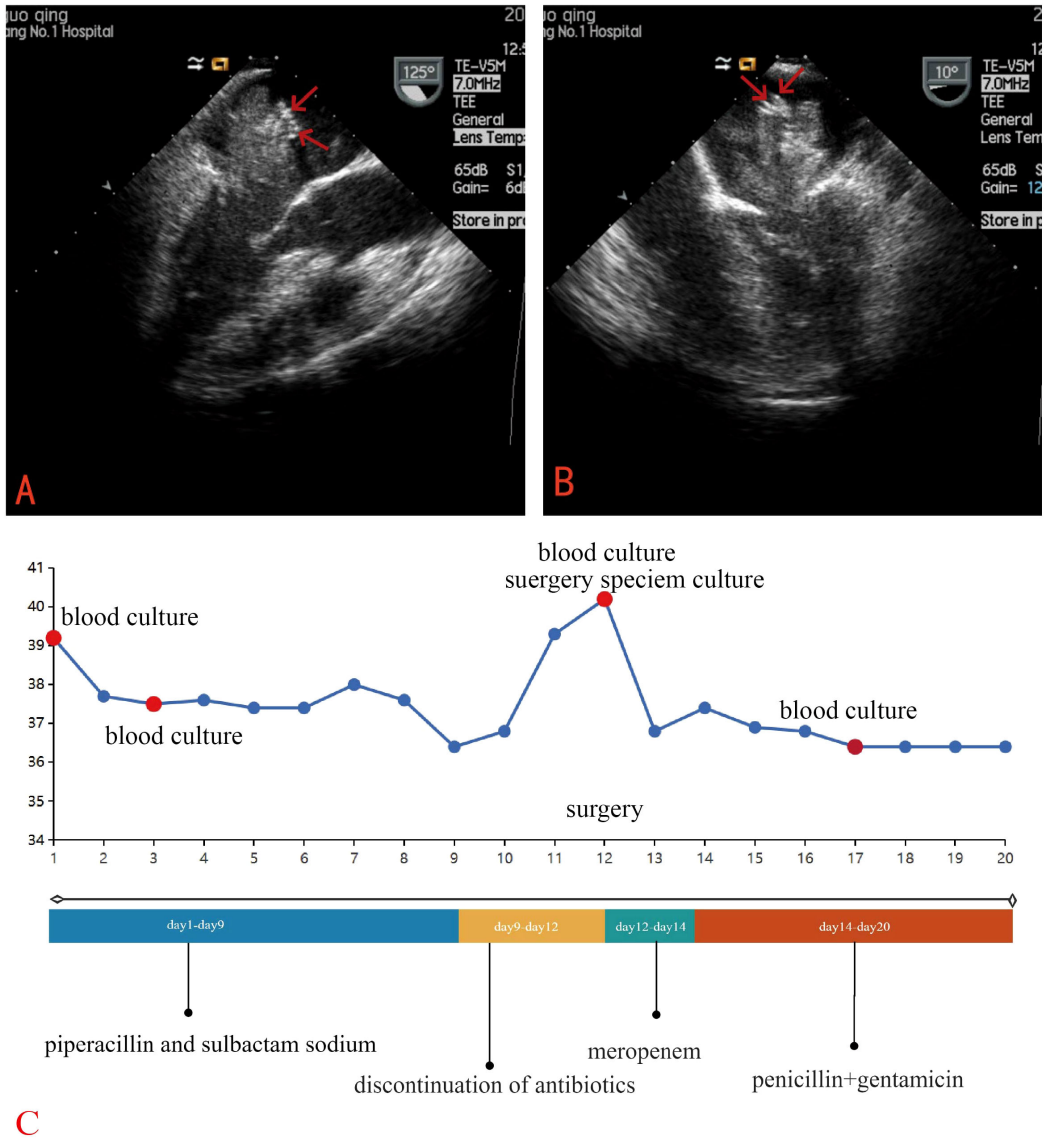
TEE revealed an elongated, irregular mass originating from the fossa ovalis in the left atrium, with a base of approximately 9 mm and dimensions of approximately 7 cm × 3 cm and punctate echogenic foci observed on the surface of the tumor (red arrow). **(A)** Long-axis view of the left ventricle **(B)** Four-chamber view **(C)** Clinical Timeline.

Laboratory tests revealed a raised white blood cell count of 12.58×10^9/L, with 83% polymorphonuclear cells. The hemoglobin concentration was 10.2 g/dL, and the hematocrit was 30.8%. The fasting blood glucose level was 9.73 mmol/L. Serum albumin and C-reactive protein (CRP) levels were 31.7 g/L and 16.10 mg/dL, respectively. Urine analysis showed glucose 2+ and protein 1 +. Chest computed tomography (CT) and electrocardiograms were normal. Aortic and coronary CT angiography (CTA) indicated a space-occupying lesion in the left atrium and a filling defect at the intersection of the abdominal aorta and bilateral common iliac arteries, suggestive of thrombosis (**Figure 1**). Transthoracic echocardiography (TTE) and TEE revealed an elongated, irregular mass originating from the fossa ovalis in the left atrium, with a base of approximately 9 mm and dimensions of approximately 7 cm × 3 cm. The tumor prolapsed into the left ventricle during diastole, causing mitral valve obstruction. TEE revealed punctate non-shadowing echogenic foci without comet tail artifact on the surface of the tumor (**Figure 2**; **Supplementary Video 1**). These findings were consistent with atrial myxoma. Additionally, the echocardiographic findings included left atrial dilation, mild to moderate tricuspid regurgitation, tachycardia, and an estimated ejection fraction of 55% in the left ventricle.

**Figure 3:**
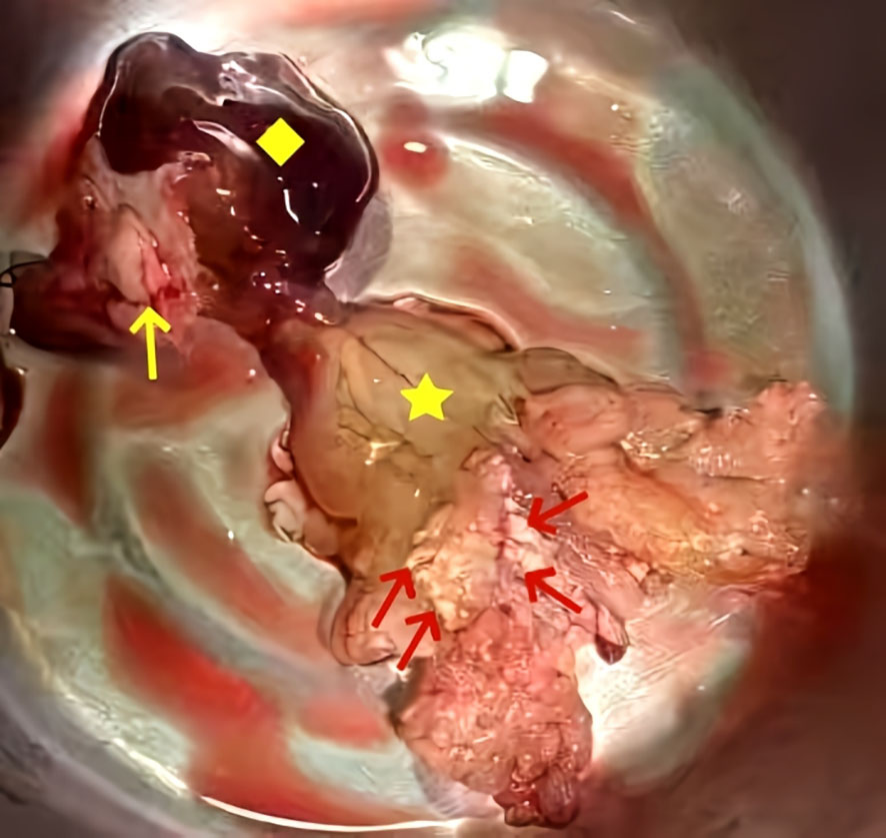
Gross examination revealed a pedunculated, lobulated, dark-red, gelatinous mass (yellow square) was observed attached to the interatrial septum (yellow arrow), with a tail-like portion exhibiting a yellow, villous tumor (five-pointed star) with an irregular surface and vegetations (red arrows).

Empirical antibiotic therapy with piperacillin and sulbactam sodium was initiated due to the patient’s presentation of fever, cough, frequent urination, along with elevated inflammatory markers, suggesting a likely respiratory or urinary tract infection. On the third day after admission, blood cultures showed the growth of Gram-positive bacilli, which was regarded as a contaminant. A repeat blood culture was negative for bacterial growth. The patient’s body temperature gradually returned to normal. Given the likelihood that the fever was caused by the cardiac myxoma, antibiotics were discontinued after the patient was transferred to the cardiothoracic surgery department. However, following the discontinuation of antibiotics, the patient’s body temperature rose again to 39°C. The patient underwent left atrial tumor resection under cardiopulmonary bypass on the third day after the transfer. Intraoperative exploration revealed a pedunculated mass located in the fossa ovalis in the left atrium. The tumor was a lobulated, dark-red, gelatinous mass attached to the interatrial septum, with a tail-like portion exhibiting a yellow, villous mass. Multiple small vegetations were visible on the surface of the tumor (**Figure 3**). The tumor and a portion of the interatrial septum were resected.

Postoperatively, the patient was managed with intravenous meropenem (1 g every 8 hours) due to the suspicion of infective endocarditis (IE). On the second postoperative day, cultures of both blood and the vegetation of the surgery specimen yielded *Streptococcus gordonii*, which was sensitive to penicillin, vancomycin, ampicillin, ceftriaxone, cefotaxime, cefoxitin, and erythromycin. Infectious disease experts recommended treatment with penicillin (3.2 million units every 6 hours) and gentamicin. (80 mg every 12 hours). Postoperative mNGS of the patient’s blood detected both *Streptococcus gordonii* and *Epstein-Barr* virus. A transthoracic echocardiogram (TTE) performed on the fourth postoperative day showed no residual mass.

**Figure 4:**
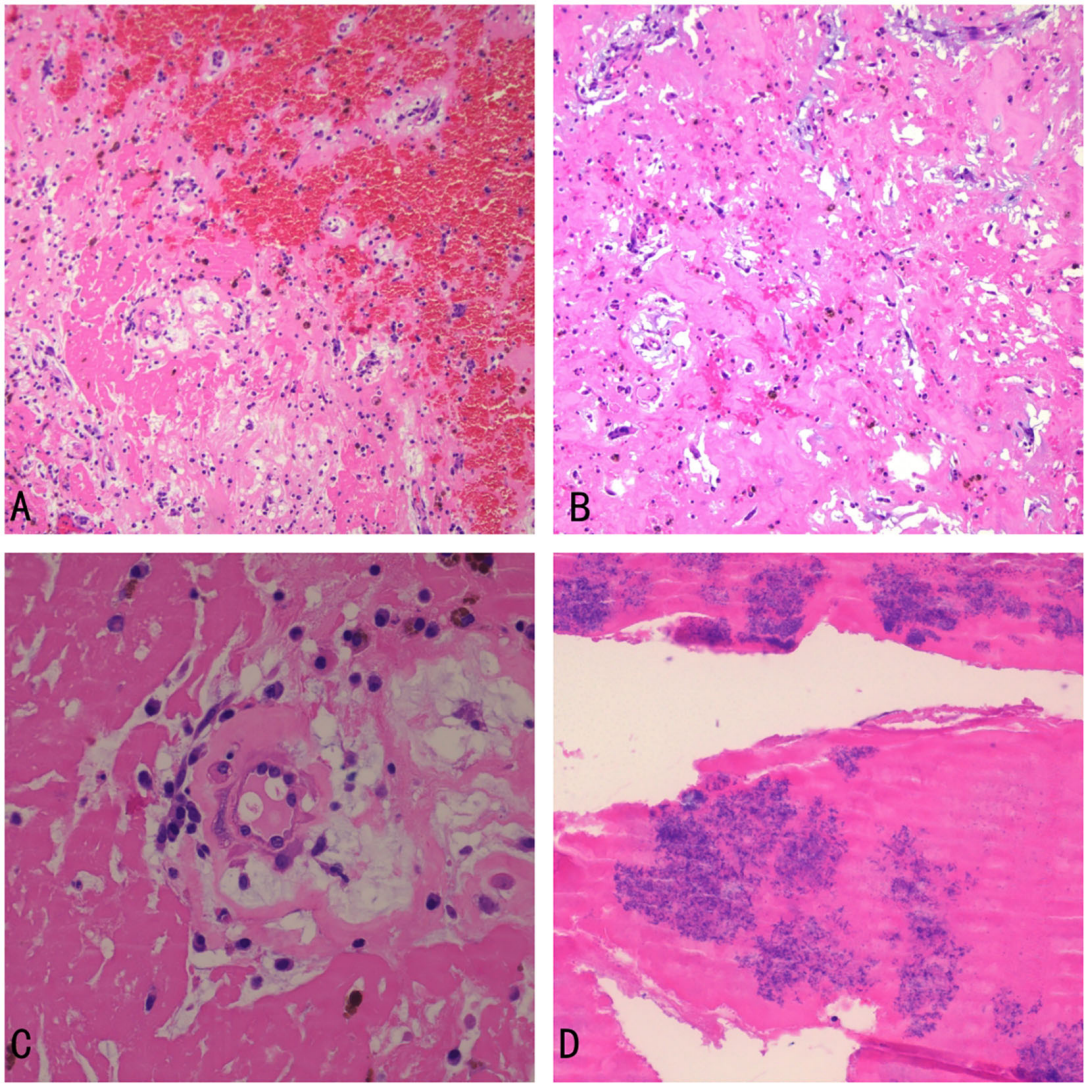
Histopathological examination **(A)** the myxoma with hemorrhage (×100) **(B)** spindle-shaped cells, and stellate cells scattered on the background of a myxomatous matrix (eosin stain, ×100) **(C)** the myxoma (×400) **(D)** Bacterial colonies and fibrin on the surface of the myxoma (×400).

Histopathological examination of the surgical specimen showed spindle-shaped cells, and stellate cells scattered on the background of a myxomatous matrix. The tumor surface was covered with fibrin and bacterial masses (**Figure 4**). These findings were compatible with a diagnosis of infected cardiac myxoma.

Postoperative blood cultures were negative. The patient was prescribed a 1-month course of penicillin and gentamicin. The patient had an uneventful postoperative recovery. Additionally, considering the presence of an abdominal aortic thrombus, continued anticoagulation therapy with aspirin will be provided after discharge.

## Discussion

Cardiac myxomas are uncommon neoplasms. Comprehensive reviews of infected cardiac myxomas were conducted by Revankar & Clark in 1998 and Shi-Min Yuan in 2015, compiling a total of 40 and 39 cases, respectively (5, 6). These reviews provided detailed characterizations of the clinical profiles associated with this rare condition. Their analyses revealed that while there is no definitive distinction between infected and non-infected cardiac myxomas, infected myxomas tend to be associated with more pronounced fever-related symptoms and a heightened risk of embolic complications. Recent dental procedures, recent infections, history of invasive surgery, and immunocompromised status are risk factors for infection of cardiac myxoma. We conducted a comprehensive literature search for all cases reported between 2015 and 2025. Using the MeSH terms “infection”, “myxoma”, “endocarditis”, “blood culture” and “bacteremia”, we performed a full-text search of the PubMed database and identified 11 relevant references (3, 7–16). Our findings are consistent with the previously reported cases in symptoms, risk factors, and complication (**Table 1**).

**Table 1:** Infected cardiac myxoma described in PubMed.

Serial	Year	Author	Sex	Year	Microorganism	Diagnostic means	Location of myxoma	Surgery	Risk factor	Complication	Outcome	Symptom	Time of antibiotic	References
1	2022	José M	Male	63	Fusobacterium nucleatum	TTE, TEE	LA	yes	diabetes mellitus	splenic abscesses	survival	fever, sweating, epigastric discomfort, weight loss	6 weeks	(7)
2	2018	Gerald Paul Fitzgerald	male	23	Streptococcus virdans	TTE, TEE	LA	yes	none	none	survival	Low fever, anorexia, weight loss, non-productive cough, night sweats, fatigue and general malaise.	6 weeks	(8)
3	2022	Coutinho	Female	33	Haemophilus spp	TTE	MV	yes	none	splenic abscesses, middle cerebral artery mycotic aneurysm	survival	fever, sweating, shortness of breath, weight loss	4 weeks	(9)
4	2023	Shi A Kim,	woman	39	Haemophilus parainfluenzae	TTE, TEE	LA	yes	none	none	survival	fever, chills, myalgia, headache, general pain	3 weeks	(10)
5	2019	Neil Patel	Male	43	*Staphylococcus aureus*	TTE, TEE	LV	yes	intravenous Heroin	cerebral infarction	survival	shortness of breath, weakness	4 weeks	(11)
6	2019	Matthew J. Peters	male	34	Streptococcus parasanguinis	TEE	LA	yes	none	middle cerebral artery mycotic aneurysm	survival	a feeling of pressure on the chest, symptoms in the left arm	6 weeks	(12)
7	2022	Masi Javeed	male	70	Escherichia coli	TTE, TEE	RA	yes	post bilateral femoral artery stents, diabetes mellitus	none	survival	Shortness of breath	6 weeks	(13)
8	2021	Jayaweera	male	46	Kodamaea ohmeri	TEE	TV	yes	a dental procedure	none	survival	fever, malaise, tiredness	6 weeks	(3)
9	2023	Kawabori	female	60	Streptococcus vestibularis	TEE	LA	yes	none	cerebral infarction	survival	fever	6weeks	(14)
10	2019	Masashi Kawabori	male	55	Staphylococcus epidermidis	TTE	LA	yes	none	splenic infarct	survival	malaise, fever, weight loss	6 weeks	(15)
11	2021	Takashi Yamamoto	female	72	Streptococcus mitis	TEE	RA	yes	a dental procedure	septic pulmonary emboli	survival	bilateral purpura, leg edema, general fatigue, weight loss	4 weeks	(16)

LA, Left Atrium; RA, Right Atrium; MV, Mitral Valve; TV, Tricuspid Valve; LV, Left Ventricle.

Fever is observed in approximately 20% of patients with non-infected cardiac myxomas (17), whereas the proportion rises significantly to 92%-97.3% in those with infected myxomas (5, 6). Although infective cases commonly present with elevated white blood cell counts (76.9%), anemia (92.9%), and increased erythrocyte sedimentation rate (ESR) and CRP (6). Non-infected cardiac myxomas with prominent systemic symptoms may also exhibit abnormal inflammatory markers (18). Consequently, fever and abnormal inflammatory markers alone cannot reliably distinguish between infected and non-infected myxomas.

In this case, the patient’s prior use of antibiotics before blood culture resulted in a negative blood culture, further complicating the diagnostic process. However, the observation that the patient’s fever was suppressed by antibiotics and recurred upon their discontinuation strongly suggested the possibility of a concomitant infection. Additionally, mNGS and PCR techniques offer significant advantages in diagnosing culture-negative IE, effectively improving the detection rate of pathogens (19).

While TEE is nearly 100% sensitive for detecting cardiac myxomas, differentiating the imaging characteristics of infected and non-infected cardiac myxomas remains challenging. In this case, a retrospective review of TEE revealed intrasolid punctate nonshadowing echogenic foci without comet tail artifact on the surface of the tumor, which were interpreted as ultrasonic manifestations of tiny, nodular vegetations that were not detected by TTE. A finger-like projecting structure seen attached to the mass is considered to be TEE manifestation of a vegetation on the tumor (20). This highlights the indispensable role of TEE in identifying infections in cardiac myxomas, particularly its enhanced capability in detecting tiny vegetations.

Systemic embolism is a common complication in patients with left atrial myxoma, with potential embolism sites including the brain, coronary arteries, aorta, kidneys, spleen, extremities, and pulmonary arteries (6). The incidence of embolism is significantly higher in infected myxomas compared to non-infected myxomas (5, 21, 22). Although this patient did not exhibit overt embolic symptoms, he was found to have an abdominal aortic thrombosis, necessitating anticoagulation and antiplatelet therapy postoperatively. Therefore, preoperative assessments should include Doppler ultrasonography of the extremity vessels, visceral vasculature CTA and cranial CT to avoid missing cases of vascular thrombus.

*Streptococci* are the second most common cause of IE after *staphylococci*, with *streptococci viridans* accounting for approximately 30% of all *streptococcal*-related endocarditis cases (23). However, *Streptococcus gordonii*, a member of the *Streptococcus sanguinis* group, is rarely reported as a cause of IE (24–28). A study in Denmark showed a higher proportion of endocarditis among patients with sepsis caused by this bacterium, which may be related to geographical distribution or differences in bacterial flora (23). Major risk factors include oral trauma, dental procedures, and immunocompromise (24). It is likely that *Streptococcus gordonii* escapes from the oral cavity and enters sterile body sites, causing a variety of severe infections when the immune system is compromised, including IE (24), empyema in the lungs (29), septic arthritis (28), and pyogenic spondylitis (30) or spondylodiscitis (31). To date, there have been no reported cases of infected cardiac myxoma caused by *Streptococcus gordonii*. The virulence factors PadA and Hsa proteins of *Streptococcus gordonii* enable the bacterium to effectively bind to the tumor cells of cardiac myxomas and platelets, forming complex biofilms composed of bacterial-platelet-fibrin complexes. Additionally, the serine-rich glycoprotein GspB in the cell wall of S. gordonii further promotes platelet aggregation, which can lead to thrombus formation.

Epstein–Barr virus (EBV) was detected in the postoperative mNGS. However, preoperative blood tests for EBV were negative, and the number of EBV sequences and the coverage were both low in the mNGS results. This suggests that there is no active EBV infection. Moreover, there was no pathological evidence of EBV infection in the cardiac myxoma. Therefore, we conclude that the detection of EBV in the mNGS was likely a coincidental finding and not related to the pathogenesis of the infected cardiac myxoma in this case.

Currently, there is no consensus regarding the optimal timing of surgery for patients with infected cardiac myxoma. Most patients are inclined to undergo initial antimicrobial therapy to stabilize their condition (6). For those who experience embolic events and disseminated intravascular coagulation, emergency surgery is more likely to be considered (4). Based on current evidence, a 30-day course of postoperative antibiotic use is considered safe and effective (6).

In conclusion, we report a case of infected cardiac myxoma caused by S*treptococcus gordonii*. This case underscores several critical clinical lessons: cardiac myxoma patients presenting with fever should be closely monitored for potential concurrent infections. Obtaining blood cultures before initiating antibiotic therapy is crucial for accurate diagnosis. When blood cultures are negative, mNGS and PCR testing can provide valuable insights into the causative pathogens. TEE is indispensable for detecting vegetations on the surface of cardiac myxomas. Early TEE can provide critical information for diagnosis. The use of appropriate antibiotics and surgical resection of the infected myxoma, are key to improving patient outcomes. Avoiding premature discontinuation of antibiotics is also crucial to ensure complete eradication of the infection.

## Data Availability

The original contributions presented in the study are included in the article/**Supplementary Material**. Further inquiries can be directed to the corresponding author.
